# A solution to the negative effects of splenectomy during colorectal trauma and surgery: an experimental study on splenic autotransplantation to the groin area

**DOI:** 10.1186/s12893-015-0105-2

**Published:** 2015-12-18

**Authors:** Bora Karip, Metin Mestan, Özgen Işık, Metin Keskin, Kafkas Çelik, Yalın İşcan, Kemal Memişoğlu

**Affiliations:** Department of General Surgery, Fatih Sultan Mehmet Training and Research Hospital, Barajyolu Caddesi Flora Evleri, E-15 Yenisehir/Atasehir, PB 34758 Istanbul, Turkey; Department of General Surgery, Kütahya Evliya Çelebi Training and Research Hospital, Kütahya, Turkey; Department of General Surgery, Özel Acıbadem Hospital, Bursa, Turkey; Department of General Surgery, Istanbul University, Istanbul School of Medicine, Istanbul, Turkey

**Keywords:** Splenectomy, Colon wound healing, Autotransplantation, Groin, Scintigraphy

## Abstract

**Background:**

Splenectomy after combined colosplenic trauma or iatrogenic splenic injury during colorectal surgery associates with worse short- and long-term outcomes, including reduced survival in patients with colorectal cancer. Splenic autotransplantation may improve the outcomes of such patients. Omental splenic transplantation is the standard procedure but may be difficult when performing laparoscopic colorectal surgery or when total or subtotal omentectomy is required. This animal model study was performed to evaluate the impact of splenic autotransplantation to the groin area on colonic wound healing.

**Methods:**

Thirty rats were divided into three groups of ten animals. One group underwent colon anastomosis and sham splenectomy, the second underwent colon anastomosis and splenectomy, and the third underwent colon anastomosis, splenectomy, and intramuscular autotransplantation of the spleen. On postoperative day 7, anastomotic healing was evaluated by measuring bursting pressure and hydroxyproline levels. The third group was subjected to scintigraphy before sacrifice to assess whether the transplant was functional.

**Results:**

The mortality rates of the sham, splenectomized, and transplanted animals were 0 %, 30 %, and 20 %, respectively: the splenectomized animals had significantly lower mean bursting pressures than the other two groups (*p* = 0.002). The mean hydroxyproline levels of the three groups were 467.4, 335.3, and 412.7 mg hydroxyproline/g protein, respectively (*p =* 0.0856). Nine of the ten transplanted animals (90 %) had splenic activity on scintigraphy.

**Conclusions:**

Splenectomy impaired the healing of the colonic anastomosis. This effect was largely reversed by splenic autotransplantation. Intramuscular autotransplantation to the groin area appears to be feasible and effective.

## Background

While the overall rate of surgical infection in patients with colon injuries is 25 %, it is worsened by concomitant splenic injury [1]. For example, Blackwood et al. showed that after splenectomy alone and colon injury alone, the intraabdominal sepsis rates are 5.7 % and 8.9 %, respectively. However, this rate increases to 46.7 % when there is a combined spleen-colon injury that requires splenectomy [2]. These observations support the notion that splenic salvage should be performed in cases of spleen injuries that accompany colon injuries [2, 3].

In elective colorectal surgery, there is an increased risk of iatrogenic splenic injury, especially during mobilization of the splenic flexura [4]. The retrospective study by Mettke et al. showed that of 46,682 patients who underwent operative treatment for colorectal cancer, 640 (1.4 %) received iatrogenic injuries of the spleen. While most of these could be repaired, splenectomy was necessary in 127 patients (0.3 % of the total population). The patients who underwent splenic repair had significantly lower morbidity and mortality rates than the patients who underwent splenectomy [5]. A retrospective analysis by Holubar et al. showed that even when multiple attempts to salvage the spleen by splenorraphy were made in cases of iatrogenic injury of the spleen after colectomy, most (70 %) still ended in splenectomy [6].

If splenectomy is necessary, the only way to preserve immunological spleen function is autotransplantation. The most popular type of transplantation is omental seeding. It has been shown that this approach is both safe and effective [7]. However, it is not the preferred treatment of choice in emergency or elective colorectal surgery. Moreover, the removal of the omentum after certain types of colorectal procedures may limit omental seeding of the splenic tissue. In such cases, an alternative approach has not yet been forthcoming.

In the present animal model study, we examined the effect of splenectomy on colonic wound healing and assessed whether intramuscular splenic autotransplantation was feasible and improved colon anastomosis healing.

## Methods

This experimental study was approved by the institutional ethics committee of Marmara University, Istanbul (document no: 47.2009.mar).

Thirty female Wistar-Albino rats weighing between 200–250 g were included in the study. After 6 h of fasting, general anesthesia was provided by intramuscular injection of 35 mg of ketamine HCl and 5 mg of xylazine HCl per kilogram of body weight. The abdominal midline was prepped with 70 % alcohol and a 5 cm standard midline incision was performed. The colon was transected 4 cm proximal of the anus with a pair of scissors.

**Fig. 1:**
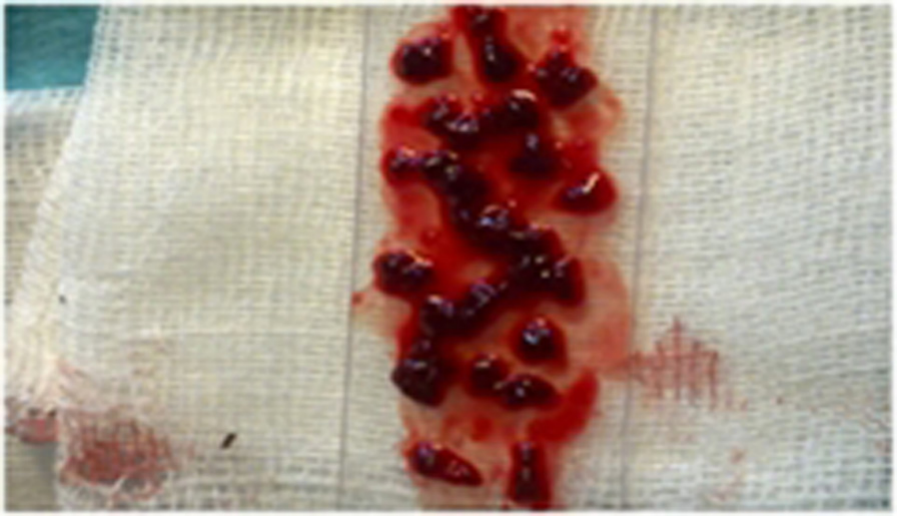
Splenic fragments prepared for transplantation into the groin area

**Fig. 2:**
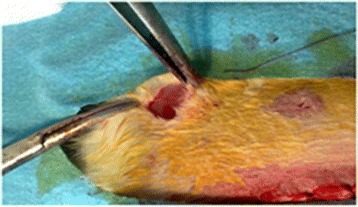
Preparation of the groin area for autotransplantation in the biceps muscle

The 30 rats were divided into three groups of ten animals. In the sham group, the spleen was mobilized, prepared for splenectomy, and then replaced into its original position in the abdomen. Thereafter, colon anastomosis was performed in a continuous fashion with 6/0 polyglactin (Vicryl, Ethicon Endo-Surgery, Cincinnati, USA). The abdomen was then filled with 10 ml of warm saline and 2 ml of air was given through a catheter into the rectum for the air-bubble test. The midline incision was closed after it was confirmed that the colon anastomosis was airtight. Closure of laparotomy was performed on two planes (the aponeurotic plane and the skin) by using continuous 3/0 polyglactin (Vicryl, Ethicon Endo-Surgery, Cincinnati, USA) suture. The splenectomy group underwent splenectomy in conjunction with colon anastomosis. For the splenectomy, the splenic vein and artery were ligated by using 4/0 polyglactin (Vicryl, Ethicon Endo-Surgery, Cincinnati, USA) suture. In the transplantation group, the rats underwent splenectomy, after which the spleen tissue was sliced into 2–3 mm sized pieces (Fig. 1) and a pouch between the two heads of the right biceps femoris muscle was created by using a clamp (Fig. 2). The spleen slices, which comprised more than 50 % of the total splenic tissue, were placed in the pouch. After colon anastomosis, the muscle fascia and the skin incision were closed by using continuous 4/0 polyglactin (Vicryl, Ethicon Endo-Surgery, Cincinnati, USA) suture. The rats were then kept in individual cages under controlled temperature (24–26 °C) and light (12 day hours followed by 12 night hours) conditions, and fed with standard rat diet.

The intent was to sacrifice the rats 7 days after surgery. Some of the animals died before this time point and underwent autopsy to determine the reason. These rats were then replaced with similarly treated rats to ensure that the three groups each consisted of ten animals that survived to postoperative day 7.

### Bursting pressure measurement

After sacrifice, the colon was transected 1.5 cm proximal and 1.5 cm distal of the anastomosis. This segment was removed together with the surrounding adhesions and organs to prevent introducing damage to the colon segment that would result in loss of airtightness. One end of the colon segment was ligated while a catheter was secured to the other end. The catheter was connected to a pressure transducer and an infusion pump that insufflated the colon lumen with a constant air pressure (200 ml/h). The pressure values were recorded by a computer. The peak value that was recorded was deemed the bursting pressure (expressed in mmHg).

### Hydroxyproline measurement

After the biomechanical measurement, the colon segments were separated from their adhesions and subjected to a spectrophotometric assay (DU 800, Beckman Coulter Inc., California, USA) to quantify the bowel hydroxyproline levels. The values were expressed as mg of hydroxyproline value per gram of protein.

### Scintigraphic study

The transplantation group underwent Tc-99m scintigraphy on postoperative day 7 prior to sacrifice. The rats were immobilized and sedated during the procedure by 3 mg/kg intramuscular diazepam. After complete sedation was achieved, technetium-99m tin colloids (500 μCi) were injected intravenously into the tail vein. When scintigraphy showed that a rat did not have a visible autotransplanted spleen, the rat was removed from the study and not replaced.

### Statistical analysis

All statistical analyses were performed by using SPSS Advanced Statistics version 20.0 (SPSS Inc, Chicago, Illinois). The three groups were compared in terms of bursting pressure and hydroxyproline measurements by using the Kruskal-Wallis test, In cases where statistical significance was found, the Mann–Whitney test was used for pairwise comparisons. *P* values of <0.05 were considered to indicate statistical significance.

## Results

**Fig. 3:**
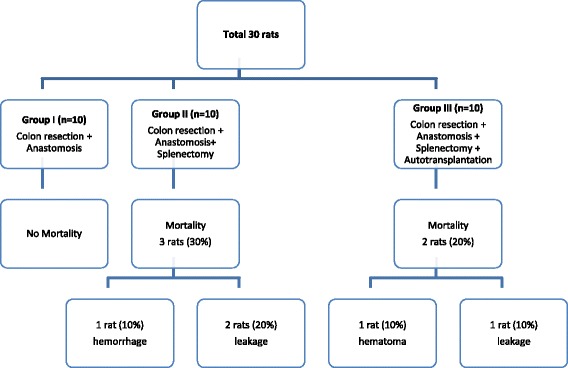
Mortality and autopsy results of the sham control, splenectomized, and autotransplanted animals

Three splenectomized and two transplanted rats died before postoperative day 7. None of the sham control rats died (Fig. 3). Thus, the mortality rates of the sham, splenectomy, and transplantation groups were 0 %, 30 %, and 20 %, respectively. One of the splenectomized rats died on postoperative day 1 due to hemorrhage from the pedicle of the spleen. The other two splenectomized rats died on postoperative days 3 and 5 as a result of anastomotic leakage and intraabdominal abcess. The two transplanted rats died on postoperative days 2 and 6. The rat that died earlier had a hematoma at the spleen autotransplant field but a satisfactory colon anastomosis. However, the other rat had an anastomotic leak.

**Fig. 4:**
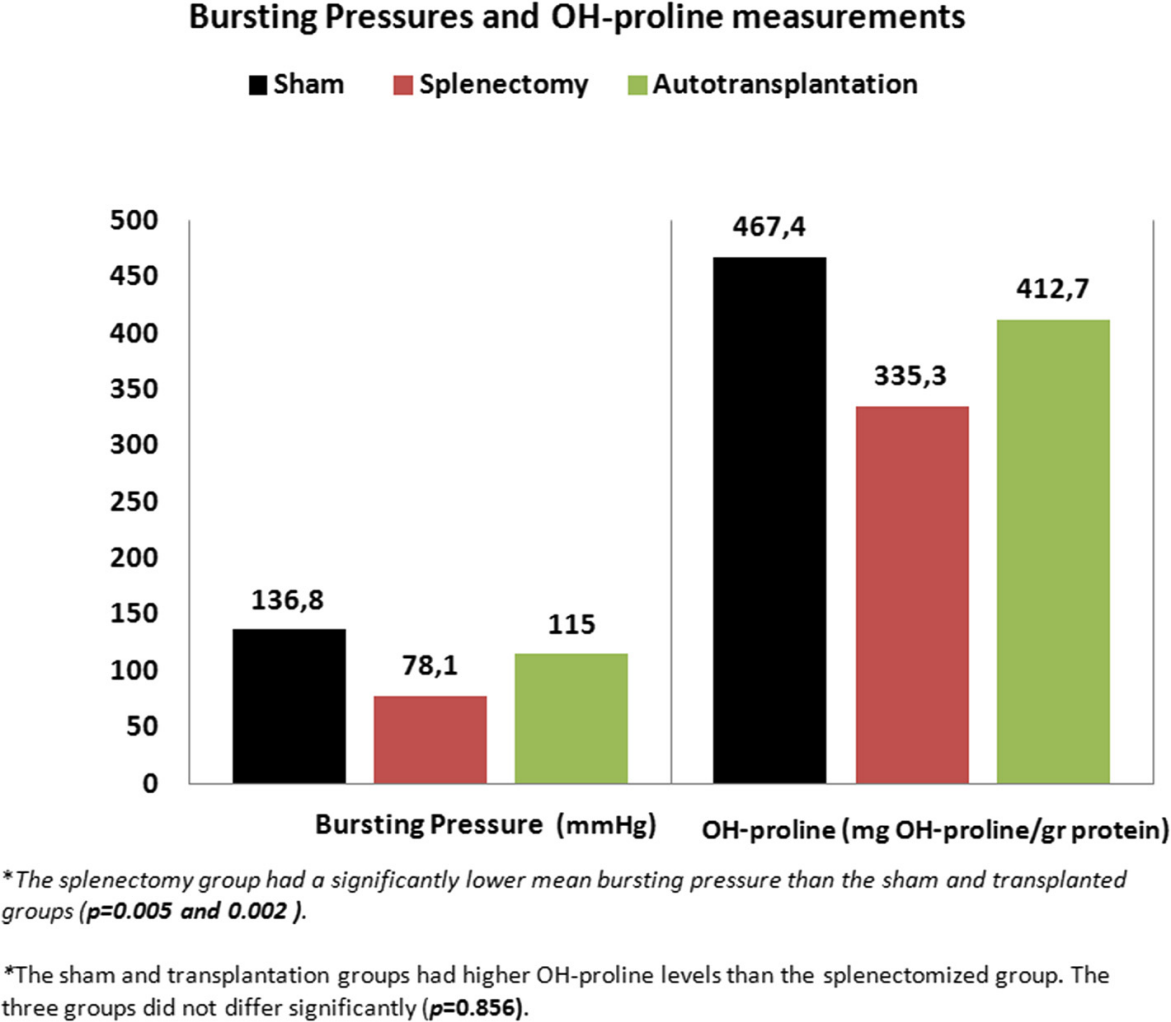
Bursting pressures and hydroxyproline scores of the sham, splenectomized, and autotransplanted animals

The mean bursting pressures of the sham, splenectomy, and transplantation groups were 136.8 ± 50.5, 78.1 ± 19.43, and 115.03 ± 31.49 mmHg, respectively (Fig. 4). The splenectomy group had a significantly lower mean bursting pressure than the sham and transplanted groups (*p* = 0.005 and 0.002). The sham and transplantation groups did not differ significantly in terms of this variable (*p* = 0.911).

The mean anastomotic hydroxyproline levels of the sham, splenectomy, and transplantation groups were 467.4, 335.3, and 412.7 mg hydoxyproline/g protein, respectively. Although the sham and transplantation groups tended to have higher hydroxyproline levels than the splenectomized group, the three groups did not differ significantly in terms of this variable (*p* = 0.856).

**Fig. 5:**
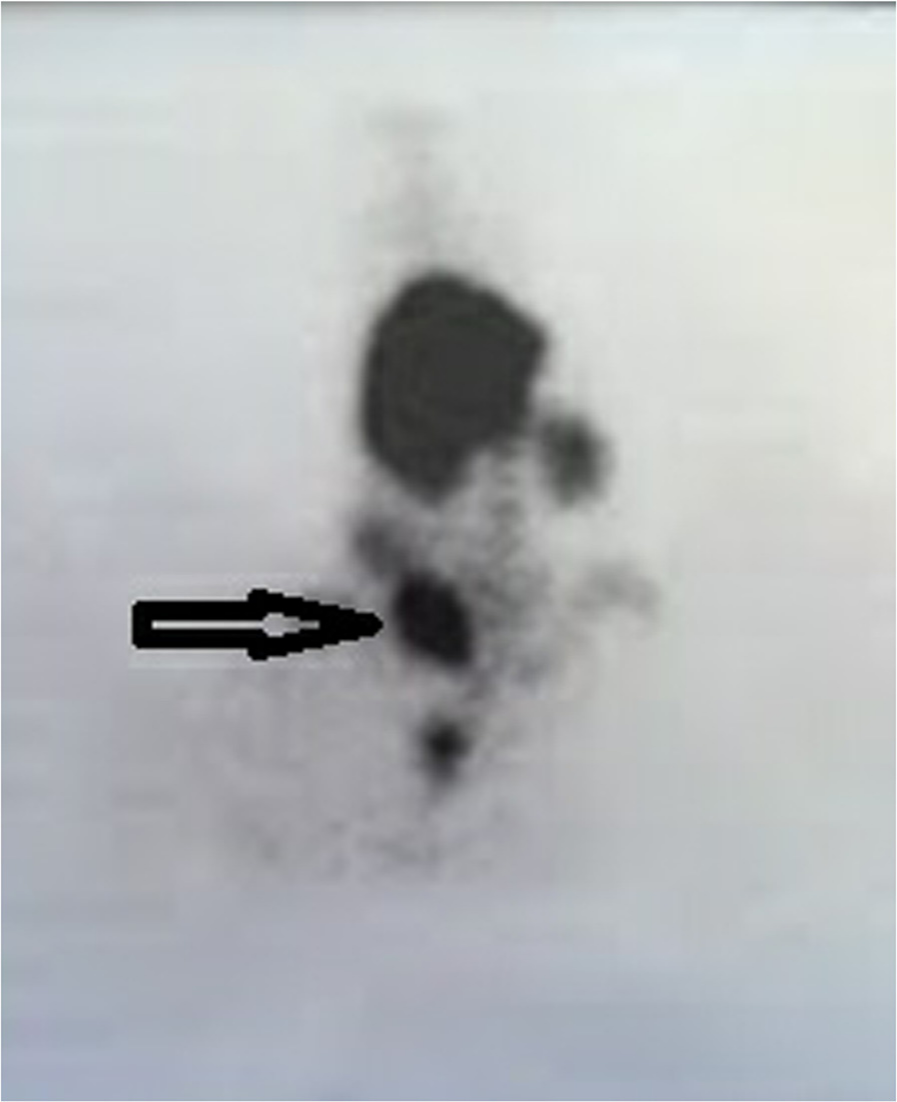
Scintigraphy of the autotransplanted rats just before sacrifice on postoperative day 7. The arrow shows the hot spot in the right groin area of a rat during Tc-99m-labeled scintigraphy

Of the ten transplanted rats, nine had detectable autotransplanted spleens, as determined by scintigraphy (Fig. 5). Thus, the scintigraphic effectiveness of the transplantation procedure was 90 %.

## Discussion

This experimental study showed that compared to the sham-operated or autotransplantation groups, the splenectomy group had significantly lower bursting pressure on postoperative day 7. The splenectomy groups also tended to have lower hydroxyproline levels than the other two groups, although these differences did not achieve statistical significance. Since hydroxyproline levels are a marker of collagen synthesis, which is essential in wound healing, it appears that the splenectomy impaired the colon anastomotic healing. This impairment was largely reversed in the splenectomized animals that underwent splenic autotransplantation to the groin area.

Until World War I, almost all colon injuries were fatal. Subsequent advances in surgery in the middle of the twentieth century caused the mortality rates to drop by 70 % [8]. At present, the use of antibiotics, fluid replacement, blood transfusions, and colostomies means that only 5 % of patients with colon injuries die; moreover, the postoperative complication rate is 15–50 % [9–11].

Colon injuries are accompanied with splenic trauma in 10–22 % of cases [3, 12]. The study of Blackwood et al. on patients with splenic injury (*n* = 58), colon injury (*n* = 90), and combined colosplenic injury (*n* = 13) showed that the intraabdominal sepsis rate that required reoperation was 5.7 % in the splenectomy group, 8.9 % in the colon repair group, and 46.7 % in the combined injury group [2].

Elective colorectal surgery also poses a risk of splenic injury. The first case of such incidental splenectomy was described in 1949 by Quan and Castleman [13]. Thereafter, in 1984, Langevin et al. mentioned that colorectal operations associate with a risk of splenic injury and splenectomy [4]. Recently, a large retrospective study of 46,682 patients who underwent surgery for colorectal cancer surgery revealed that 1.4 % received splenic injury; of these, 127 cases resulted in splenectomy (0.3 % of the total population). In addition, the patients who underwent spleen repair were found to have a significantly lower mortality rate than the patients who had to undergo splenectomy (4.7 % *vs.* 11.8 %) [5]. Several other studies have reported similar results [14–19]. These observations support the notion that attempts should be made to salvage the spleen after concurrent colon and splenic injuries or after incidental splenic injury in elective colorectal surgery.

Clinicians have long been seeking alternative methods to treat overwhelming post-splenectomy infection (OPSI) in splenectomized patients [20, 21]. Developments in surgical techniques and abdominal imaging have increasingly allowed the spleen to be salvaged after blunt trauma and iatrogenic injuries [22]. However, splenorraphy is usually unsuccessful in combined colosplenic injuries [6]. In cases where the splenic injury cannot be managed without splenectomy, splenic autotransplantation may be recommended. Indeed, the study by Moore et al. on splenic autotransplantation in 23 patients with combined colosplenic injuries showed that only one patient developed postoperative infectious complications. In addition, they showed that peritoneal contamination was not a contraindication to performing splenic autotransplantation [23].

Several animal model studies have assessed the effect of splenic autotransplantation on sepsis after splenectomy. Alves et al. reported that after splenic autotransplantation in mice, there is neovascularization between the transplanted spleen and the main vessels on the third day after transplantation; the blood supply of the splenic fragments is eventually provided in a centripetal manner by the splenic, short gastric, mesenteric, and gastroepiploic arteries [24]. Patel et al. showed that, in splenectomized rabbits, autotransplantation with small slices of spleen in the peritoneal cavity improves the clearance of pneumococci from the blood [25]. Marques et al. reported that splenic autotransplantation prevents *Escherichia coli* sepsis in rats. Moreover, they showed that the splenic slices transplanted in the greater omentum acquire a normal splenic microscopic and macroscopic architecture, and retain bacterial phagocyte function [26]. Several other studies showed that splenic autotransplantation preserves bacterial clearance capability [27–29]. However, the autotransplanted spleen is smaller and does not have all of its original functional capacity [30].

It is not clear whether splenic autotransplantation has the same effects in humans because it is unethical to subject splenectomized humans to the experiments needed, namely, bacterial clearance measurements or relaparatomy to assess the regenerated splenic mass [7]. However, Patel et al. found that splenic autotransplantation in their four cases associated with preservation of platelet counts, peripheral blood smear results, immunoglobulin M levels, and complement component 3 levels. Moreover, scintigraphy scans at postoperative week 8 revealed the presence of functioning splenic tissue [31]. In 2002, Zhang et al. used partial splenic autotransplantation in patients who had undergone the modified Sugiura operation. In the control group, splenectomy was performed, while in the study group, partial splenic autotransplantation into the retroperitoneal space was performed. Two months after the operation, spleen function, in terms of serum tuftsin and serum IgM levels, was stable in the autotransplantation group [32].

At present, the most common splenic autotransplantation method is the inoculation of splenic tissue into pouches created in the omentum [33, 34]. When Iinuma et al. transplanted 25, 50, 100, 200, and 300 mg of splenic tissue in the omental pouches, intramuscular field, or intraperitoneally, they found that the omental pouch is the most effective location for splenic autotransplantation and that at least 50 % of the splenic tissue should be transplanted [35]. However, omental splenic autotransplantation during colorectal surgery is not feasible when total or subtotal omentectomy must be performed. Moreover, although it has been shown that laparoscopic colorectal surgery associates with a lower splenic injury rate, possibly because of better visualization [19], if a splenic injury does occur during a laparoscopic procedure, it can be technically challenging to prepare and autotransplant the spleen in the abdominal cavity. Furthermore, splenic autotransplantation into the omentum is associated with intestinal obstruction and the development of intraabdominal abscess [23, 36–38]. Thus, the intramuscular splenic autotransplantation method can sometimes be more technically advantageous compared to autotransplantation in abdominal and other sites. Our study showed that 90 % of the intramuscular transplanted animals exhibited functioning splenic tissue on scintigraphy, which indicates that this approach is feasible.

The present study showed for the first time that splenectomy impaired colon anastomic healing after colorectal surgery, and that intramuscular splenic transplantation improved the healing to nearly normal levels. While wound healing after colorectal surgery has been studied in various conditions, the effect of splenectomy on colon anastomotic healing has not been assessed previously. Although gastrointestinal wounds heal in a similar fashion to wounds in other tissues, there are some differences. In particular, under normal conditions, wounds in the intestinal wall develop tensional strength earlier than wounds in skin [39]. This may reflect the fact that the smooth muscle cells in the intestinal wall serve as an extra source of collagen synthesis [40]. How splenectomy impedes colon anastomosis healing is unclear. However, it may reflect the fact that splenectomy depletes T cell numbers. This in turn may reduce the ability of T cells to induce fibroblastic activity, namely, collagen synthesis and wound healing [41]. This notion is supported by the study of Barbul et al., who showed that lymphokines promote the proliferation, migration, and protein synthesis of fibroblasts *in vitro* [42]. Moreover, Werbin et al. showed that although fibroblast activity was normal 1 week after rats were splenectomized, there was a marked depletion in this activity 1 month post-splenectomy. They also reported that when the rats underwent splenic autotransplantation, the spleen function and wound healing normalized [43]. In addition, Ertekin et al. reported that compared to sham operation, splenectomy associates with impaired colonic wound healing and reduced hydroxyproline levels and tensile strength [44]. These observations together led Karip et al. to conclude recently that autotransplantation to the omentum should be considered during colorectal surgery as it may lower the risk of infection and provide possible long-term infectious and immunological benefits [45].

## Conclusions

Splenectomy concurrent with colectomy impaired anastomotic healing. This effect was largely reversed by splenic autotransplantation to the groin area. Since this approach was feasible in our study, we conclude that if splenectomy must be performed during open or laparoscopic colorectal surgery, inguinal splenic autotransplantation may be a useful alternative to omental splenic transplantation in emergency or elective procedures.

## References

[1] Dawes LG, Aprahamian C, Condon RE, Malangoni MA (1986). The risk of infection after colon injury. Surgery.

[2] Blackwood JM, Hurd T, Suval W, Machiedo GW (1988). Intra-abdominal infection following combined spleen-colon trauma. Am Surg.

[3] Kemmeter PR, Hoedema RE, Foote JA, Scholten DJ (2001). Concomitant blunt enteric injuries with the injuries of the liver and the spleen: a dilemma for trauma surgeons. Am Surg.

[4] Langevin JM, Rothenberger DA, Goldberg SM (1984). Accidental splenic injury during surgical treatment of the colon and rectum. Surg Gynecol Obstet.

[5] Mettke R, Schmidt A, Wolff S, Koch A, Ptok H, Lippert H (2012). Spleen injuries during colorectal carcinoma surgery. Effect on the early postoperative result. Chirurg.

[6] Holubar SD, Wang JK, Wolff BG, Nagorney DM, Dozois EJ, Cima RR (2009). Splenic salvage after intraoperative splenic injury during colectomy. Arch Surg.

[7] Pisters PWT, Pachter HL (1994). Autologous splenic transplantation for splenic trauma. Ann Surg.

[8] Maxwell RA, Fabian TC (2003). Current management of colon trauma. World J Surg.

[9] Hudolin T, Hudolin I (2005). The role of primary repair fot colonic injuries in wartime. Br J Surg.

[10] Demetriades D, Murray JA, Chan L, Ordoñez C, Bowley D, Nagy KK (2001). Penetrating colon injuries requiring resection: diversion or primary anastomosis? An AAST prospective multicenter study. J Trauma.

[11] Sasaki LS, Mittal V, Allaben RD (1994). Primary repair of colonic injuries; a retrospective analysis. Am Surg.

[12] Huizinga WK, Baker LW (1993). The influence of splenectomy on infective morbidity after colonic and splenic injuries. Eur J Surg.

[13] Quan S, Castleman B (1949). Splenic-vein thrombosis following transthoracic gastrectomy and incidental splenectomy. N Engl J Med.

[14] Wakeman CJ, Dobbs BR, Frizelle FA, Bissett IP, Dennett ER, Hill AG (2008). The impact of splenectomy on outcome after resection for colorectal cancer: a multicenter, nested, paired cohort study. Dis Colon Rectum.

[15] Davis CJ, Ilstrup DM, Pemberton JH (1988). Influence of splenectomy on survival rate of patients with colorectal cancer. Am J Surg.

[16] Varty PP, Linehan IP, Boulos PB (1993). Does concurrent splenectomy at colorectal cancer resection influence survival?. Dis Colon Rectum.

[17] Konstadoulakis MM, Kymionis GD, Leandros E, Ricaniadis N, Manouras A, Krespis E (1999). Longterm effect of splenectomy on patients operated on for cancer of the left colon: a retrospective study. Eur J Surg.

[18] Steinert R, Depel M, Schmidt A, Ptok H, Meyer F, Wolff S (2014). Iatrogenic splenic injuries in surgery of colorectal carcinoma: Impact on the oncological long-term of outcome. Chirurg.

[19] Kastenmeier A, Ludwig KA (2012). Splenic injury during colon surgery: a matter of technique?. Arch Surg.

[20] Morris DH, Bullock FD (1919). The importance of the spleen in resistance to infection. Ann Surg.

[21] King H, Shumacker HB (1952). Splenic studies: 1. Susceptibility to infection after splenectomy performed in infancy. Ann Surg.

[22] Shackford SR, Molin M (1990). Management of splenic injuries. Surg Clin North Am.

[23] Moore FA, Moore EE, Moore GE, Millikan JS (1984). Risk of splenic salvage after trauma. Analysis of 200 adults. Am J Surg.

[24] Alves HJ, Viana G, Magalhães MM, Arantes RM, Coelho PM, Cunha-Melo JR (1999). Kinetics of neovascularisation of splenic autotransplants in mice. J Anat.

[25] Patel JM, Williams JS, Naim JO, Hinshaw JR (1986). The effect of site and technique of splenic tissue reimplantation on pneumococcal clearance from the blood. J Pediatr Surg.

[26] Marques RG, Petroianu Y, Coelho JM (2003). Bacterial phagocytosis by macrophage of autogenous splenic implants. Braz J Biol.

[27] Brown EJ, Hosea SW, Frank MM (1981). The role of the spleen in experimental pneumococcal bacteremia. J Clin Invest.

[28] Moxon ER, Schwartz AD (1980). Heterotropic splenic autotransplantation in the prevention of Haemophilus influenza meningitis and fatal sepsis in Sprage-Dawley rats. Blood.

[29] Livingston CD, Levine BA, Sirinek KR (1983). Intraperitoneal splenic autotransplantation. Protection afforded in a naturally occuring epidemic murine mycoplasmosis. Arch Surg.

[30] Smith E, De Young N J, Drew PA (2003). Decreased phagocytic capacity of autotransplanted splenic tissue. ANZ J Surg.

[31] Patel J, Williams JS, Shmigel B, Hinshaw JR (1981). Preservation of splenic function by autotransplantation of traumatized spleen in man. Surgery.

[32] Zhang H, Chen J, Kaiser GM, Mapudengo O, Zhang J, Exton MS (2002). The value of partial splenic autotransplantation in patients with portal hypertension: a prospective randomized study. Arch Surg.

[33] Velcek VT, Jongco B, Shaftan GW (1982). Posttraumatic splenic replantation in children. J Pediatr Surg.

[34] Durig M, Landermann RMA, Harder F (1984). Lymphocyte subsets in human peripheral blood after splenectomy and autotransplantation of splenic tissue. J Lab Clin Med.

[35] Iinuma H, Okinaga K, Sato S, Tomioka M, Matsumoto K (1992). Optimal site and amount of splenic tissue for autotransplantation. J Surg Res.

[36] Büyükünal C, Danismend D, Yeker D (1987). Spleen-saving procedures in pediatric splenic trauma. Br J Surg.

[37] Bem C, Echun D (1991). Regeneration of the spleen and splenic autotransplantation (letter). Br J Surg.

[38] Nielsen JL, Sakso P, Sorensen FH, Hansen HH (1984). Demonstration of splenic functions following splenectomy and autologous spleen implantation. Acta Chir Scand.

[39] Cronin K, Jackson D, Dunphy JE (1968). Changing bursting strength and collagen content of the healing colon. Surg Gynecol Obstet.

[40] Graham MF, Drucker DEM, Diegelmann RF, Elson CO (1987). Collagen synthesis by human intestinal smooth muscle cells in culture. Gastroenterology.

[41] Peterson JM, Barbul A, Breslin RJ, Wasserkrug HL, Efron G (1987). Significance of T lymphocytes in wound healing. Surgery.

[42] Barbul A, Knud-Hansen J, Wasserkrug BA, Efron G (1986). Interleukin 2 enhances wound healing in rats. J Surg Res.

[43] Werbin H (1983). The spleen and wound healing. Eur Surg Res.

[44] Ertekin C, Dilege S, Kurtoğlu M, Genç S, Çevikbaş U (1991). Splenektominin yara iyileşmesi üzerine etkisinin deneysel olarak incelenmesi. Ulusal Cerrahi Dergisi.

[45] Karip B, Işcan Y, Keskin M, Kapan M, Ağca B, Altun H, et al. Splenic autotransplantation is safe in colorectal surgery. Kolon Rektum Hast Derg. 2014;24:125–32.

